# Dairy-Protein-Based Aggregates as Additives Enriched with Tart Cherry Polyphenols and Flavor Compounds

**DOI:** 10.3390/foods12112104

**Published:** 2023-05-24

**Authors:** Mirela Kopjar, Ivana Buljeta, Ina Ćorković, Vanja Kelemen, Anita Pichler, Ivana Ivić, Josip Šimunović

**Affiliations:** 1 Faculty of Food Technology, Josip Juraj Strossmayer University, F. Kuhača 18, 31000 Osijek, Croatia; ivana.buljeta@ptfos.hr (I.B.); ina.corkovic@ptfos.hr (I.Ć.); anita.pichler@ptfos.hr (A.P.); ivana.ivic@ptfos.hr (I.I.); 2 Teaching Institute of Public Health Osijek-Baranja County, Franje Krežme 1, 31000 Osijek, Croatia; vanya.kelemen@gmail.com; 3 Department of Food, Bioprocessing and Nutrition Sciences, North Carolina State University, Raleigh, NC 27695-7624, USA; simun@ncsu.edu

**Keywords:** dairy proteins, tart cherry polyphenols, tart cherry flavor compounds, antioxidant activity

## Abstract

Nowadays, the development of innovative food products with positive health effects is on the rise. Consequently, the aim of this study was a formulation of aggregates based on tart cherry juice and dairy protein matrix to investigate whether different amounts (2% and 6%) of protein matrix have an impact on the adsorption of polyphenols as well as on the adsorption of flavor compounds. Formulated aggregates were investigated through high-performance liquid chromatography, spectrophotometric methods, gas chromatography and Fourier transform infrared spectrometry. The obtained results revealed that with an increase in the amount of protein matrix used for the formulation of aggregates, a decrease in the adsorption of polyphenols occurred, and, consequently, the antioxidant activity of the formulated aggregates was lower. The amount of protein matrix additionally affected the adsorption of flavor compounds; thus the formulated aggregates differed in their flavor profiles in comparison with tart cherry juice. Adsorption of both phenolic and flavor compounds caused changes in the protein structure, as proven by recording IR spectra. Formulated dairy-protein-based aggregates could be used as additives which are enriched with tart cherry polyphenols and flavor compounds.

## 1. Introduction

Lately, there has been a great need for the development of innovative food products possessing many positive health benefits, such as antioxidative, antidiabetic and anti-inflammatory effects [1]. The modern, busy lifestyle has a large impact on eating habits which can lead to a negative impact on health. Quite often, food is related to the fulfillment of desire, and a balance between pleasure and health has to be found [2]. Due to these reasons, functional foods are gaining more attention from consumers; however, their attractiveness cannot be neglected. Cherries are a rich resource of polyphenols and other bioactive food components, including fibers, vitamin C, carotenoids and potassium. There are two main types of cherries, sweet cherries (*Prunus avium* L.) and tart cherries (*Prunus cerasus* L.). Most of the sweet cherries are consumed fresh, while 97% of tart cherries are processed into different products for direct consumption or baking or cooking [3]. Natural polyphenols present in fruits and vegetables have poor stability, can be easily degraded and are sensitive to light, heat and oxygen. The application of polyphenols in food products is also limited as a result of their unpleasant taste and formation of off-color [1]. From this perspective, there is a need for the development of alternatives that minimize the nutritional and sensory losses while achieving greater stability of polyphenols [4]. Considering the structural and functional varieties of proteins, different approaches of exploiting proteins for the delivery of bioactives have been recommended [1]. It has been reported that protein-based aggregates provide a protective effect on polyphenols. For instance, the antioxidant activity of proteins increased after modification with certain polyphenols [5]. However, interactions between proteins and polyphenols are various and complicated since they both show high sensitivity to the environment [6]. Larger polyphenols have been reported to form complexes with dairy proteins. This binding can influence the electron donation capacity of polyphenols through a reduction in the number of hydroxyl groups available in the mixture. Caseins are major phosphoproteins of mammalian milk and belong to the group of unstructured proteins with unfolded structure and low hydrophobicity under native conditions [7]. Cyanidin-3-rutinoside, cyanidin-3-glucosyl-rutinoside and cyanidin-3-glucoside are anthocyanins detected in tart cherries, while the most abundant of the phenolic acids is chlorogenic acid [8]. It has been reported that there is a strong relationship between anthocyanin intake and anti-inflammatory activity, protection against neurological diseases and reduction of heart disease, obesity and diabetes. In the food industry, anthocyanins have the potential to be used as natural dyes, as synthetic dyes can cause adverse effects [4]. As consumers’ awareness of the significance and advantages of a healthy diet is on the rise, the use of tart cherry in the development of functional foods is necessary [9]. In the review of Lila et al. [10], the importance of protein/polyphenol particles and their possible utilization in the food industry, as well as the bioaccessibility of bioactive polyphenolic compounds and edible proteins were explored. In the food industry, depending on their properties, proteins have a broad range of applications. They can be used as emulsifiers, stabilizers of foams and colloid systems and film-forming polymers [11,12]. In the past decade, their utilization as microencapsulating carriers of different active compounds has been the focus of many studies, and an especially distinct segment is the encapsulation of phenolics. In this way, stability of phenolics can be achieved and bioactive protein-based aggregates can be formulated [13,14,15,16,17,18]. Usually, during the formulation of protein-based aggregates, when fruit material is used as a resource of phenolic compounds, the adsorption of flavor compounds has been neglected. There have been studies proving that during the formulation of functional additives based on proteins or fibers next to phenolics, flavor compounds were also adsorbed on the applied carrier [19,20,21,22]. Another important factor affecting the overall acceptance of the food products on the market is the characteristic flavor. The overall flavor profile of fruits is affected by the equilibrium between volatile compounds and their concentrations. Tart cherries are known for their specific and pleasant flavor. Different organic compounds such as carbonyls, alcohols, acids, esters and terpenes make up the flavor of tart cherries [23]. During the formulation and preparation of food products including functional additives, changes in flavor composition are often a result of the impact of processing conditions which can cause different chemical changes in flavor compounds and other food constituents, and, additionally, processing conditions can lead to interactions between them. Such protein-based aggregates can be utilized for modulation of the phenolic profile, flavor profile, color and antioxidant potential of different food products to which they are added.

The aim of this research was to formulate aggregates from tart cherry juice and dairy protein matrix to investigate whether different amounts (2% and 6%) of protein matrix have an impact on the adsorption of polyphenols adsorption as well as on the adsorption of flavor compounds. High-performance liquid chromatography (HPLC) was applied for the identification and quantification of polyphenols present in the samples, while the flavor profile was evaluated using gas chromatography with mass spectrometry (GC-MS) analysis. Color parameters were measured, and Fourier transform infrared spectroscopy (FTIR-ATR) screening of aggregates was performed. Additionally, antioxidant activity was determined spectrophotometrically using different assays.

## 2. Materials and Methods

### 2.1. Chemicals

Dairy proteins were obtained from Ingredia Functional (Arras, France). Methanol used for analysis was HPLC grade and it was obtained from J.T. Baker (Deventer, The Netherlands). Orthophosphoric acid was also HPLC grade (>85%) and it was purchased from Fisher Scientific (Loughborough, UK). 4-dimethyl-amino-cinnamaldehyd, trolox, 2,2′-azino-bis(3-ethylbenzothiazoline-6-sulfonic acid) diammonium salt, 2,2-diphenyl-1-picrylhydrazil and standards for HPLC analysis (rutin, quercetin, chlorogenic acid, p-coumaric acid, (−)-epicatechin) were products of Sigma-Aldrich (St. Louis, MO, USA). The standard of cyanidin-3-rutinoside was from Phytolab GmbH (Vestenbergsgreuth, Germany) and the standard of neochlorogenic acid was the product of Extrasynthese (Genay, France). Folin-Ciocalteu reagent and potassium persulfate were products of Kemika (Zagreb, Croatia). Neocuproine, cupric chloride and 2,4,6-tri(2-pyridyl)-s-triazine (TPTZ) were products of Acros Organic (Geel, Belgium). Sodium carbonate was bought from T.T.T. (Sveta Nedelja, Croatia) and hydrochloric acid (37%) from Carlo Erba Reagents (Sabadell, Spain).

### 2.2. Preparation of Tart Cherry/Dairy Protein Aggregates

Two different percentages (2% and 6%) of dairy protein matrix (86% of protein on dry matter with 80% of casein micelles) were used for the preparation of aggregates with tart cherry juice. Mixing of protein matrix and juice was performed on a magnetic stirrer (Stuart US152, Buch and Holm, Hervel, Denmark) for 15 min at ambient temperature. Using centrifugation for 15 min at 4000 rpm, the precipitate was separated from the supernatant. After that, the frozen precipitate was freeze-dried using a freeze dryer, Alpha 1–4 (Christ, Osterode am Harz, Germany). The freeze-drying conditions were previously specified [20].

### 2.3. Extraction of Polyphenols from Tart Cherry/Dairy Protein Aggregates

Approximately 0.4 g of sample was extracted with 15 mL of extraction solvent (HCl:methanol 1:99). The extraction was conducted for 24 h at ambient temperature. Obtained extracts were evaluated spectrophotometrically and used for HPLC analysis.

### 2.4. Determination of Total Polyphenol Content, Proanthocyanidin Content and Anthocyanin Content in Formulated Aggregates

Total polyphenol content was assessed through the utilization of a spectrophotometric method [24]. Extract (0.2 mL) was mixed with demineralized water (1.8 mL), Folin–Ciocalteu reagent (10 mL) and 7.5% sodium carbonate solution (8 mL). The reaction mixture was kept in the dark for 120 min and the absorbance was read at a wavelength of 765 nm using a UV/Vis spectrophotometer (Cary 60 UV-Vis, Agilent Technologies, Santa Clara, CA, USA). Gallic acid was used for the expression of results; its calibration curve was constructed, and the obtained results were expressed as g of gallic acid per kg of tart cherry/dairy protein aggregates (g GAE/g). Each sample was measured in triplicate.

The DMAC (4-dimethyl-amino-cinnamaldehyd) method by Prior et al. [25] was used for the determination of proanthocyanidin content. Prepared samples were measured in triplicate and the absorbance was determined at 640 nm. A procyanidin B2 was used for the expression of results; its calibration curve was constructed, and the obtained results were expressed as mg of procyanidin B2 per g of tart cherry/dairy protein aggregates (mg PB2E/g).

Anthocyanin concentration was evaluated with the pH differential method that was previously published by Giusti et al. [26]. Two different buffers were made—0.025 M KCl at pH 1 and 0.4 M sodium acetate at pH 4.5—and 2.8 mL of each buffer was mixed with 0.2 mL of extract. Prepared mixtures were put in a dark place for 15 min. The absorbance was measured at two different wavelengths (515 and 700 nm). Then, the final absorbance of the sample was determined by the following equation:

(1) $$A={\left(A_{515}-A_{700}\right)}_{\mathrm{pH}\;1}-{\left(A_{515}-A_{700}\right)}_{\mathrm{pH}\;4.5}$$

To determine the anthocyanin concentration, the following equation was used:

(2) $$\mathrm{Monomeric}\;\mathrm{anthocyanins}=\left({A\times M}_{W}\times \mathrm{DF}\times 1000\right)/\left(\varepsilon \times l\right)$$

where MW is 449.2 g/mol (the molecular weight of cyanidin-3-glucoside), DF is the dilution factor, ε is 26,900 L/mol cm (the molar absorptivity) and l is the 1 cm cuvette length. Finally, the monomeric anthocyanins concentration was expressed as mg of cyanidin-3-glucoside per gram of tart cherry/dairy protein aggregates (mg cyanidin-3-glucoside/g).

### 2.5. Antioxidant Activity (DPPH, FRAP, CUPRAC, ABTS Assays) of Formulated Aggregates

Antioxidant activity of tart cherry/dairy protein aggregates was achieved spectrophotometrically using DPPH, FRAP, CUPRAC and ABTS assays. All measurements were performed using a UV/Vis spectrophotometer. For all assays, calibration curves were made using Trolox. The results were expressed as µmol of Trolox per 100 g of aggregates (µmol TE/100 g). All aggregates were analyzed in triplicate. The DPPH method was previously described in the study by Brand-Williams et al. [27]. Briefly, DPPH solution at a concentration of 0.5 mM was made, and 3 mL of that solution was added to the extract. After incubation that lasted for 15 min in a dark place, the absorbance was read at 517 nm. To determine ferric reducing ability, FRAP reagent was added to 0.2 mL of the extract, and, after it was incubated for 30 min, the absorbance was read at 593 nm. This method was described in detail elsewhere [28]. The protocol for the CUPRAC assay was previously published by Apak et al. [29]. Briefly, 1 mL of CuCl_2_ (10 mM), 1 mL of neocuproine (7.5 mM), 1 mL of ammonium acetate buffer (1 M, pH 7.0), extract and distilled water at a total volume of 1.1 mL were mixed and put in a dark place for 30 min, and then the absorbance was read at 450 nm. A slightly modified method from Arnao et al. [30] was used to carry out the ABTS assay. Extracts were mixed with ABTS reagent. After 95 min of incubation, the absorbance was read at 734 nm.

### 2.6. Evaluation of Polyphenols by High-Performance Liquid Chromatography (HPLC) in Formulated Aggregates

Concentrations of individual polyphenols in tart cherry/dairy protein aggregates were determined using an Agilent HPLC system 1260 Infinity II (Agilent Technology, Santa Clara, CA, USA). The system consisted of a quaternary pump, a vial sampler and a column (Poroshell 120 EC C-18; 4.6 × 100 mm, 2.7 µm). Mobile phase A was 0.1% orthophosphoric acid, and mobile phas B was 100% methanol. The gradient used was previously published in the study by Buljeta et al. [31]. Before injection into the system, extracts were filtered through PTFE filters (pore size 0.2 μm). The flow rate was set at 1 mL/min and the injected volume was 5 µL. Each extract was injected two times. UV/Vis spectra were recorded in the range from 190 to 600 nm. Peaks were identified by comparing the UV-Vis spectra of peaks in extracts and retention times. The calibration curves were constructed for all standards of polyphenols (cyanidin-3-rutinoside, neochlorogenic acid, chlorogenic acid, *p*-coumaric acid, rutin, quercetin and (−)-epicatechin) and calibration curve linearity was confirmed by r^2^ > 0.99. Cyanidin-3-glucosyl-rutinoside was identified according to the literature [32] and quantified using the cyanidin-3-rutinoside curve. The concentration of *p*-coumaric acid derivate was calculated using the calibration curve constructed for *p*-coumaric acid. Concentrations of identified polyphenols were expressed as mg of polyphenol per kg of tart cherry/protein aggregates (mg/kg).

### 2.7. Color Parameters of Formulated Aggregates

A CR-400 chromometer (Konica Minolta, Inc., Osaka, Japan) was used to record the following parameters of color: L* represents lightness (0 is black and 100 is white); a* represents redness (+) or greenness (−); b* indicates yellowness (+) or blueness (−); C* denotes saturation of color and °h represents the hue angle [33]. Formulated aggregates were measured three times. The color difference between the protein matrix and formulated aggregates (ΔE*) was calculated by the equation:

(3) $$\Delta E^{*}=\sqrt{{\left({\Delta L}^{*}\right)}^{2}+{\left({\Delta a}^{*}\right)}^{2}+{\left({\Delta b}^{*}\right)}^{2}}$$

### 2.8. Evaluation of Flavour Compounds

Flavor compounds were evaluated in tart cherry juice, dairy protein matrix and formulated aggregates. Flavor compounds from samples were extracted using solid-phase microextraction (SPME). A quantity of 0.3 g of each aggregate, together with 4.7 g of water and 1 g of NaCl, were put into the vial. SPME fiber coated with divinylbenzene/carboxen/polydimethylsiloxane (DVB/CAR/PDMS) sorbent (50/30 µm, StableFlex™, Supelco, Bellefonte, PA, USA) was applied for the extraction of flavor compounds. The method was described in detail in the study of Vukoja et al. [22]. Confirmation of the compounds was achieved by comparison of their mass spectra with the National Institute of Standards and Technology mass spectral database (NIST, East Amwell Township, NJ, USA) and through retention time and retention index. Two replicates were conducted for each aggregate. Myrtenol was used as an internal standard for quantification, and the results were expressed as µg/kg.

### 2.9. Fourier Transform Infrared with Attenuated Total Reflection (FTIR-ATR) Spectroscopy Analysis

IR spectra were collected by screening samples on an FTIR-ATR instrument (Cary 630, Agilent, Santa Clara, CA, USA), and evaluation was performed using the software MicroLab Expert (Agilent, Santa Clara, CA, USA). IR spectra of formulated aggregates and protein matrix were screened from 4000 cm^−1^ to 600 cm^−1^ with 32 scans at a resolution of 8 cm^−1^.

### 2.10. Statistical Analysis

Statistical analysis of all obtained data was carried out with the software STATISTICA 13.1 (StatSoft Inc., Tulsa, OK, USA), through the analysis of variance (ANOVA) and Fisher’s least significant difference test, where the significance was defined at *p* < 0.05. Additionally, a principal component analysis was carried out.

## 3. Results

### 3.1. Phenolic Compounds and Antioxidant Activity of Formulated Aggregates

The total phenolic, proanthocyanidin and monomeric anthocyanin contents, along with the antioxidant activities of the formulated aggregates, are presented in Table 1. Comparing parameters that were evaluated on formulated aggregates, it can be concluded that the amount of protein matrix used for complexation highly affected the adsorption of polyphenols and consequently affected antioxidant activity. Aggregates formulated with 2% of the protein matrix had higher values of all evaluated parameters than aggregates with 6% of the protein matrix. Even though the increase in protein matrix during complexation was threefold, the decrease of adsorption didn’t follow that trend. Total phenolic contents of aggregates were 874.93 mg/100 g and 369.78 mg/100 g for Cas2%_TC and Cas6%_TC, respectively. Aggregates with 2% of protein matrix had a concentration of proanthocyanidins 619.45 mg/100 g and aggregates with 6% of protein matrixhad a concentration of 273.18 mg/100 g. In both cases, the concentration was 2.3 times higher in Cas2%_TC than in Cas6%_TC. A lower decrease with an increase of protein amount was observed for monomeric anthocyanins, from 137.52 mg/100 g to 78.75 mg/100 g, indicating that Cas2%_TC had 1.7 times higher concentration than Cas6%_TC. Additionally, values of antioxidant activities that were determined by the DPPH, ABTS, CUPRAC and FRAP methods also did not follow a trend of threefold increase. For DPPH antioxidant activity, Cas2%_TC had values 1.2 times higher than those of Cas6%_TC, while for all other methods, the antioxidant activity of Cas2%_TC was around 2 times higher than that of Cas6%_TC. These results indicate that methods are based on different mechanisms of action but also that individual phenolic compounds contribute differently to antioxidant activity.

Specific phenolic compounds were also evaluated in formulated aggregates and are presented in Table 2 (additionally Figures S1–S4). Two anthocyanins were identified, cyanidin-3-glucosyl-rutinoside and cyanidin-3-rutinoside. As it was determined for monomeric anthocyanins, both individual anthocyanins were determined in higher concentration in aggregates with the lower amount (2%) of the protein matrix, 504.66 mg/kg vs. 783.09 mg/kg for aggregates with 6% of protein matrix. These aggregates had 1.1 times higher concentrations of these anthocyanins than aggregates with 6% of protein matrix (458.72 mg/kg and 675.5 mg/kg, respectively). Two flavonoids were identified, rutin and quercetin, and their adsorption was also affected by the amount of protein matrix. The same pattern as for anthocyanins was determined. Cas2%_TC had 1.2 times higher concentration of both flavonoids than Cas6%_TC. Rutin was evaluated in concentrations of 259.79 mg/kg and 209.02 mg/kg in Cas2%_TC and Cas6%_TC, respectively, while quercetin for the same samples was evaluated in lower concentrations, 89.57 mg/kg and 79.33 mg/kg, respectively. Chlorogenic acid, neochlorogenic acid and *p*-coumaric acid and its derivate were identified from phenolic acids. All of these acids were determined in higher concentrations in Cas2%_TC: 470.23 mg/kg, 352.90 mg/kg, 559.40 mg/kg and 927.04 mg/kg, respectively. In this aggregate, these concentrations were 2.3 higher for chlorogenic and neochlorogenic acids and 1.3 times higher for *p*-coumaric acid and its derivate than in Cas6%_TC (186.44 mg/kg, 159.88 mg/kg, 454.07 mg/kg and 626.51 mg/kg, respectively). Additionally, epicatechin was identified in aggregates in a concentration of 1156.63 mg/kg in Cas2%_TC, which was 4.4 times higher than in Cas6%_TC (249.26 mg/kg).

Formulated aggregates differ in their color parameters, which are presented in Table 3. L* value (lightness) of the protein matrix decreased with the adsorption of tart cherry juice components onto it. Cas6%_TC aggregate had a significantly higher L* value than was expected since that sample contained a higher amount of protein matrix. The a* value (redness) increased upon adsorption of juice components on the protein matrix, and this increase was higher for Cas2%_TC aggregate, which is in accordance with the concentration of anthocyanins that adsorbed onto the protein matrix. Adsorption of juice components caused a decrease in b* values (yellowness) of aggregates, and this decrease was more pronounced for Cas6%_TC. The °h value decreased with the adsorption of juice components; however, there was no difference between aggregates. The C* value (saturation of color) increased, and a higher increase was evaluated for Cas6%_TC.

### 3.2. Flavor Compounds and Flavor Profile of Formulated Aggregates

The identified flavor compounds of all samples and their properties are presented in Table 4. Flavor compounds of tart cherry juice, dairy protein powder and formulated aggregates were determined by GC-MS (Table 5) and compounds were grouped into aldehydes and ketones, alcohols, terpenes and esters. Tart cherry juice is rich in flavor compounds; however, during its complexation with dairy protein powder and the formulation of aggregates, partial adsorption of fruit flavor compounds onto the protein matrix occurred. Dairy protein powder also contained some flavor compounds, so the final flavor of the formulated aggregates was the combination of the flavors of both applied ingredients. However, some flavor compounds of the initial ingredients were completely lost during the complexation and formulation of the aggregates. Additionally, it was observed that the amount of protein matrix used for complexation affected the adsorption of flavor compounds. In tart cherry juice, 13 flavor compounds from the aldehyde and ketone groups were detected; in protein powder, nine were detected; and in formulated aggregates, only six were detected. Tart cherry juice contained hexanal, 2-heptanone, heptanal, benzaldehyde, 1-octen-3-one, 6-methyl-5-hepten-2-one, nonanal, decanal, 4-propylbenzaldehyde, dodecanal, geranylacetone, myristaldehyde and hexylcinnamal. The protein matrix contained some of the same flavor compounds as tart cherry juice (hexanal, benzaldehyde, nonanal, decanal, geranylacetone, myristaldehyde and hexylcinnamal) but also two compounds that were not identified in juice (octanal and lillial). In formulated aggregates, only six compounds from this chemical group were identified. Hexanal, benzaldehyde, nonanal, decanal and geranylacetone originated from both initial ingredients and octanal and lillial from the protein matrix. Hexanal was identified in both initial ingredients and in formulated aggregates, and it was determined that its concentration in formulated aggregates was higher than in the initial ingredients. Probably, alongside the adsorption of hexanal onto the protein matrix, the transformation of other compounds into it also occurred. A similar effect was observed for octanal that originated from the protein matrix. Additionally, this compound was found in a higher concentration when a higher amount (6%) of protein matrix was used for complexation. The remaining aldehydes and ketones in the formulated aggregates were determined in lower concentrations than in the initial ingredients. Benzaldehyde, which is one of the key flavor compounds of the overall flavor of tart cherries, was identified in all samples. Comparing its adsorption on the protein matrix, it is evident that higher adsorption was achieved when a lower amount (2%) of protein matrix was used for complexation. Of the alcohols, five were identified in tart cherry juice (2-ethyl-1-hexanol, benzyl alcohol, phenethyl alcohol, decanol and perillyl alcohol) and three in the protein matrix (octen-3-ol, 2-ethyl-1-hexanol and 1-octanol). In the formulated aggregates, five compounds were identified, namely 2-ethyl-1-hexanol, benzyl alcohol, 1-octanol, decanol and perillyl alcohol, meaning that phenethyl alcohol from juice was not adsorbed on the protein matrix and that octen-3-ol was lost from the protein matrix during complexation. Next to benzaldehyde, benzyl alcohol and phenethyl alcohol are also very important compounds that contribute to the overall flavor of the tart cherry. While benzyl alcohol was adsorbed on the protein matrix (with higher adsorption observed when 2% of the protein matrix was used for complexation), phenethyl alcohol was not adsorbed at all. Terpenes are generally very important fruit flavor contributors. Twelve flavor compounds from this group were identified in tart cherry juice (D-limonene, α-terpinolene, linalool, α-campholenal, nerol, geraniol, vitispirane, eugenol, α-ionol, β-damascenone, α-ionone and β-ionone), while in protein matrix, only 3 of them (geraniol, trans-chariophyllene and β-ionone) were identified. Seven terpenes were identified in formulated aggregates, namely D-limonene, linalool, geraniol, eugenol, α-ionol, α-ionone and β-ionone, which mostly originated from juice, with the exception of geraniol. Comparing the impact of protein matrix amount during complexation, it can be concluded that all terpenes adsorbed in higher amounts when a lower amount (2%) of the matrix was used for complexation, with the exception of D-limonene. Several esters were also identified in the samples: 2-ethylhexyl acetate, ethyl benzoate and ethyl decanoate in tart cherry juice and ethyl decanoate in the protein matrix. Interestingly, none of those esters were identified in the formulated aggregates. On the contrary, probably due to the transformation of other compounds, methyl acetate was determined in aggregates with a higher concentration when 2% of protein matrix was used for complexation.

**Figure 1:**
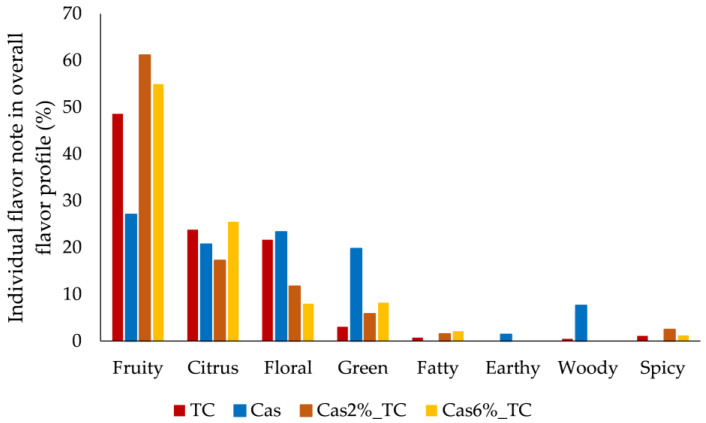
Contribution of individual flavor notes in the overall flavor profile of tart cherry juice (TC), protein matrix (Cas) and formulated aggregates (Cas2%_TC and Cas6%_TC).

Since each flavor compound is characterized by its odor descriptor and flavor compounds were detected in different concentrations in samples, this resulted in different flavor profiles of initial ingredients and formulated aggregates (Figure 1). According to odor descriptors of flavor compounds, fruity, citrus, floral, green, fatty, earthy, woody and spice flavor notes were identified in samples. In the overall flavor profile of tart cherry juice, the fruity flavor note prevailed (49%), followed by citrus and floral flavor notes (around 23%) with a slight scent of green, spicy and fatty flavor notes (<3%). The protein matrix was characterized by fruity, floral, citrus and green flavor notes, which contributed almost equally to its final flavor (27%, 23%, 21% and 20%, respectively), with a slight scent of woody and earthy flavor notes (8% and 1%, respectively). The formulated aggregates were a combination of the flavor notes of initial ingredients, and the amount of protein matrix affected the final overall profile of aggregates. Generally, the fruity flavor note prevailed in aggregates and it was more pronounced in aggregates prepared with 2% of protein matrix (61%) in contrast to 55% in aggregates prepared with 6%. The fruity flavor note was followed by the citrus one, but the opposite effect was observed with regard to the amount of protein matrix. The citrus flavor note was more pronounced in aggregates prepared with 6% of protein matrix (26%) in contrast to 17% in aggregates prepared with 2%. The floral flavor note followed the trend that was obtained for the fruity one (12% for aggregates with 2% of protein matrix and 8% for aggregates with 6%). Green, fatty and spicy flavor notes were also noted but there was no difference between aggregates with different amounts of protein matrix used for complexation (8%, 2% and 2%, respectively).

**Figure 2:**
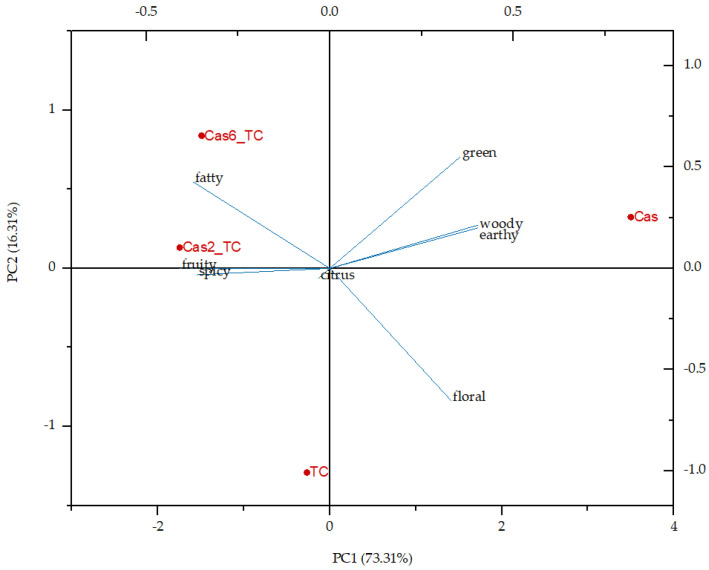
Principal component analysis of individual flavor notes in the overall flavor profile of tart cherry juice (TC), protein matrix (Cas) and formulated aggregates (Cas2_TC and Cas6_TC—aggregates with 2% and 6% of protein matrix).

Additionally, flavor notes were analyzed through principal component analysis (Figure 2). To the total variance, PC1 accounted for 73.31% and PC2 for 16.31%. The protein matrix was on the positive side of PC1 while all the other samples were on the negative side. Tart cherry juice was on the negative side of PC2 and all the other samples were on the positive side, so it can be also concluded that the initial ingredients were quite different in their flavor profiles, while the formulated aggregates were a combination of flavor notes of both initial ingredients.

### 3.3. IR Spectra of Formulated Aggregates

**Figure 3:**
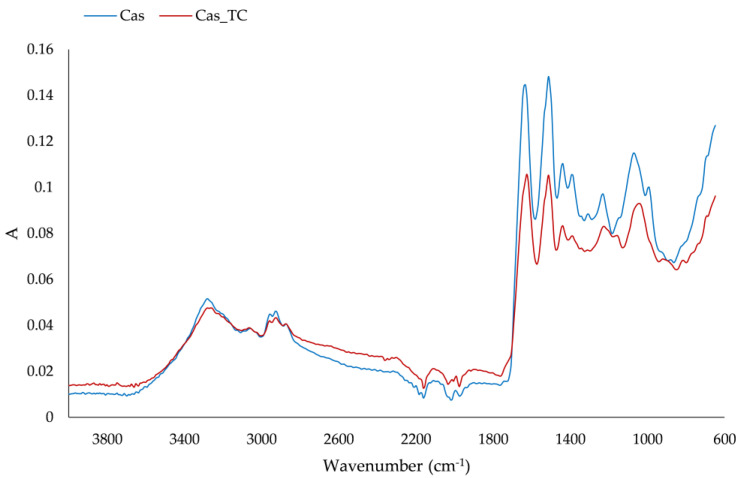
IR spectra of protein matrix (Cas) and formulated aggregate (Cas_TC).

Characteristic amide bands were defined in regions at wavenumbers of 1700–1600 cm^−1^, 1600–1500 cm^−1^, 1380–1200 cm^−1^ and 3300–3500 cm^−1^, and these were associated with amide I (C-O stretching), amide II (N-H bending and C-H stretching), amide III (N-H in plane bending coupled with C-N stretching) and amide A structures of proteins, respectively [34,35,36,37]. The IR spectra of protein matrix and formulated aggregate are presented in Figure 3. Protein powder and formulated aggregates had these characteristic amide regions; however, the intensity of bands on the formulated aggregates was lower. Changes in the amide I region, which reflect the secondary structure of the protein, were detected. The band at the protein matrix on 1640 cm^−1^ due to the adsorption of compounds from tart cherry juice shifted to 1632 cm^−1^, and, additionally, a slight shoulder formed at 1647 cm^−1^. Different secondary structures can be detected on proteins such as α-helix (1658–1650 cm^−1^), β-sheet (1640–1610 cm^−1^), β-turn (1700–1660 cm^−1^) and random coil structure (1650–1640 cm^−1^) [36]. From our IR spectra, it was apparent that the change in β-sheet occurred with a slight change in the random coil structure. Changes in amide II structure were not detected but there were changes in the amide III region. The band at 1237 cm^−1^ shifted to 1230 cm^−1^ and broadened with a slight shoulder at 1200 cm^−1^. These changes indicated changes in β-sheet [34]. Bands in the region 1060–890 cm^−1^ are mostly assigned to C–C stretching with 1080 cm^−1^, 1038 cm^−1^ and 933 cm^−1^, and related to α-helices and C–O stretching vibration [38]. We also observed a change in this region that could be associated with α-helices: the band at 1073 cm^−1^ shifted to 1051 cm^−1^ upon adsorption of tart cherry compounds. Additionally, upon adsorption of tart cherry compounds on the protein matrix, the band at 991 cm^−1^ completely disappeared and two bands, at 916 cm^−1^ and 879 cm^−1^, combined into one at 900 cm^−1^. Also, on the protein matrix, the band at 1730 cm^−1^, which was assigned to C = O, disappeared.

## 4. Discussion

Results of other studies that were conducted with the aim of creating protein/polyphenol enriched matrices proved that that type of protein matrix as well as the polyphenol source has a great impact on the adsorption of these valuable bioactives on the selected carrier. Additionally, the concentrations of the initial ingredients and the conditions during the formulation of such matrices was of great importance. Through the study on the formulation of delivery systems of raspberry phenolics based on brown rice protein matrix, it was concluded that, with a increase in the amount of protein matrix during the formulation of protein aggregates, a decrease in adsorption capacity occurred [16,17,18,20]. Our results were in accordance with those results. However, in this case, a higher decrease in adsorption capacity with an increase in protein matrix was observed for total phenolic compounds and anthocyanins. The importance of the protein source and the amount of proteins in this source have been proven in the study of the binding of cranberry polyphenols on defatted soy flour (50% of proteins), soy protein isolate (70% of proteins), hemp protein isolate (70% of proteins), medium-roast peanut flour (50% of proteins) and pea protein isolate (85% of proteins) [16]. The highest adsorption of total phenolic of cranberries was determined for hemp protein isolate, and the lowest for soy protein isolate, while for anthocyanins the highest adsorption was observed on defatted soy flour and the lowest, again for soy protein isolate [16]. In the study of the adsorption of blueberry polyphenols on defatted soybean flour in combination with white whole wheat, brown rice or corn flours, it was concluded that proteins and other insoluble components of used plant flours were included in the overall adsorption of blueberry polyphenols. More precisely, it was concluded that the protein content of the utilized flours was not linearly related with the binding efficiency for blueberry anthocyanins [18], which is in accordance with the results of this study. Therefore, the authors conclude that, in their case, carbohydrates (the major components of flours) were also involved in the overall adsorption of phenolics. Additionally, when they individually tested the mentioned carriers, they determined that defatted soy flour, with the highest content of proteins, had the highest adsorption capacity of blueberry anthocyanins, followed by wheat, rice and corn flours [18]. For our protein aggregates, it can be concluded that, through the complexation of protein matrix and tart cherry juice, when a higher amount of protein matrix was used, additional interactions (hydrogen bonding and/or hydrophobic interactions) between protein chains occurred. In this way, the number of binding sites on proteins for polyphenols decreased. On the other hand, tart cherry juice contained flavor compounds that also bonded on protein molecules, additionally diminishing the number of free binding sites on proteins. Generally, the reactivity of phenolics depends on the number of hydroxyl groups, but it also depends on their position, so this is also valid for interactions between phenolics and proteins and, additionally, the protein fraction—i.e., the free binding sites on them is very important [39,40,41]. Interactions of both a covalent and non-covalent nature are important between phenolics and proteins [42,43]. Considering non-covalent binding between phenolics and protein hydrophobic association, hydrogen bonds, electrostatic attraction and van der Waals forces can be involved. However, hydrophobic interaction and the formation of hydrogen bonds were considered the most relevant non-covalent interactions for the complexation of phenolics with proteins [44]. 

In most cases, possible additional adsorption of flavor compounds next to phenolics is neglected. However, it has been proven that during the complexation of brown rice protein with raspberry juice, both phenolics and flavor compounds from juice adsorbed onto the protein matrix [20]. The same trend was observed when cellulose was used for the encapsulation of raspberry [22] or apple fibers for blackberry juice phenolics [19,21]. Our results are in accordance with those studies. It is evident that the amount of dairy protein matrix used affected the adsorption of flavor compounds from tart cherry juice, as it has been proven for protein and fiber carriers applied for the formulation of functional additives in those studies. Also, the behavior of flavor compounds, i.e., their adsorption onto the carrier, depended on the properties of the flavor compounds and their affinity towards the carrier. Similar interactions between proteins and flavor compounds occur, as already explained for the interaction between proteins and phenolics. In this case, also, there is no universal mechanism that has been put forward as a favored one. Reversible hydrogen bonding, ionic bonding, van der Waal’s forces, and irreversible chemical binding via covalent linkages with amino and sulfhydryl groups can be possible interactions between proteins and flavor compounds [45]. To explain the complete binding mechanism between those compounds, a combination of different molecular forces/interactions as a function of the chemical structure of flavor compounds as well as matrix composition is necessary to explain, and should be taken into consideration [46,47]. Changes in the interactions of proteins with other compounds highly depend on the microenvironment of amino acid residues, hydrophobicity and the secondary structures of proteins [48]. These interactions cause differences in the concentrations of flavor compounds in samples and the ratios among them. Since each flavor compound is characterized by flavor notes, the previously mentioned factor has a high impact on overall flavor profile, which was evident from our research as well as previous ones.

As proof of the adsorption of phenolic and flavor compounds of tart cherry juice on dairy protein matrix, IR spectra of samples were recorded. Changes on the protein matrix due to different interactions between those initial ingredients were observed, as was the case in other studies dealing with the formulation of diverse protein-based microparticles. Those changes varied from minor to larger, depending on the type and properties of the adsorbed compounds and the protein matrix [20,49,50,51].

## 5. Conclusions

The aim of this research was to formulate functional protein aggregates based on dairy proteins and tart cherry juice. In most studies, additional adsorption of flavor compounds next to polyphenols from fruit sources is neglected; thus the investigation of both types of compounds on formulated aggregates was investigated. Our results showed that, next to polyphenols from tart cherry juice, flavor compounds were also adsorbed on the protein matrix, altering the protein matrix flavor profile, not only the polyphenol profile and color. Formulated dairy-protein-based aggregates can be applied for the enrichment of different products (fruit, dairy, and bakery products) with proteins, as well as polyphenols and flavor compounds. This type of additive could be beneficial in the formulation of novel innovative food products and the improvement of existing ones.

## Figures and Tables

**Table 1:** Total phenolic content (TPC), proanthocyanidin content (PAC), monomeric anthocyanins (MA) and antioxidant activity of formulated aggregates.

	Cas2%_TC	Cas6%_TC
TPC (mg/100 g)	874.93 ± 9.20 ^b^	369.78 ± 0.77 ^a^
PAC (mg/100 g)	619.45 ± 3.16 ^b^	273.18 ± 0.67 ^a^
MA (mg/100 g)	137.52 ± 0.26 ^b^	78.75 ± 0.95 ^a^
DPPH (μmol/100 g)	32.90 ± 0.47 ^b^	25.81 ± 0.38 ^a^
FRAP (μmol/100 g)	6.70 ± 0.07 ^b^	3.07 ± 0.07 ^a^
ABTS (μmol/100 g)	41.69 ± 0.08 ^b^	21.35 ± 0.11 ^a^
CUPRAC (μmol/100 g)	442.09 ± 2.39 ^b^	207.95 ± 1.41 ^a^

TC—tart cherry juice, Cas—dairy protein matrix, 2% and 6%—the amounts of protein matrix used for complexation. Values in the same row labeled with different letters (^a^,^b^) were statistically different.

**Table 2:** Individual phenolic compounds that were identified and quantified in formulated aggregates (mg/kg).

Phenolic Compounds	Cas2%_TC	Cas6%_TC
Cyanidin-3-glucosyl-rutinoside *	504.66 ± 6.21 ^b^	458.72 ± 3.81 ^a^
Cyanidin-3-rutinoside	783.09 ± 15.96 ^b^	675.45 ± 3.08 ^a^
Rutin	259.79 ± 1.75 ^b^	209.02 ± 0.74 ^a^
Quercetin	89.57 ± 1.44 ^b^	79.33 ± 0.82 ^a^
Neochlorogenic acid	352.90 ± 9.48 ^b^	159.88 ± 0.80 ^a^
Chlorogenic acid	470.23 ± 14.48 ^b^	186.44 ± 1.32 ^a^
*p*-Coumaric acid	559.40 ± 6.80 ^b^	454.07 ± 1.68 ^a^
*p*-Coumaric acid derivate **	927.04 ± 13.34 ^b^	626.51 ± 0.98 ^a^
(−)-Epicatechin	1156.63 ± 30.92 ^b^	249.26 ± 7.35 ^a^

Cas—dairy protein matrix; TC—tart cherry juice; *—tentatively identified and quantified using cyanidin-3-rutinoside calibration curve; **—tentatively identified and quantified using *p*-coumaric acid calibration curve. Values in the same row labeled with different letters (^a^,^b^) were statistically different.

**Table 3:** Parameters of color of the dairy protein matrix and formulated aggregates.

Samples	L*	a*	b*	ΔE	°h	C*
Cas	92.03 ± 0.01 ^c^	−1.47.97 ± 0.03 ^a^	12.28 ± 0.02 ^c^		96.81 ± 0.05 ^b^	12.37 ± 0.02 ^a^
Cas2%_TC	51.68 ± 0.15 ^a^	19.21 ± 0.08 ^c^	7.19 ± 0.01 ^b^	45.63	20.53 ± 0.09 ^a^	20.51 ± 0.07 ^c^
Cas6%_TC	56.11 ± 0.10 ^b^	14.79 ± 0.06 ^b^	5.47 ± 0.01 ^a^	40.01	20.29 ± 0.11 ^a^	15.77 ± 0.05 ^b^

Cas—dairy protein matrix; TC—tart cherry juice. Values in the same column labeled with different letters (^a–c^) were statistically different.

**Table 4:** Volatile compounds identified in tart cherry juice (TC), dairy protein powder (Cas) and formulated aggregates (Cas_TC).

Flavor Compounds	TC	Cas	Cas_TC	RT	RI	MW	Log P	VP	OD
Hexanal	+	+	+	5.03	800	100.16	1.78	10.88	green
2-heptanone	+	−	−	9.85	885	114.19	1.98	4.73	fruity
Heptanal	+	−	−	10.80	897	114.19	2.442	3.85	green
Benzaldehyde	+	+	+	14.61	956	106.1	1.48	1.27	fruity
1-octen-3-one	+	−	−	16.45	979.5	128.2	2.52	0.531	fruity
Octen-3-ol	−	+	−	16.50	979	128.21	2.519	0.531	earthy
6-methyl-5-hepten-2-one	+	−	−	16.90	984.98	126.2	1.90	1.277	citrus
Octanal		+	+	18.00	998	128.22	2.951	2.068	green
D-limonene	+	−	+	19.17	1017.7	136.2	4.57	0.198	citrus
2-ethyl-1-hexanol	+	+	+	19.66	1026.5	139	3.10	0.207	citrus
Benzyl alcohol	+	−	+	19.81	1029	192.3	3.20	0.008	fruity
1-octanol	−	+	+	22.22	1069.1	130.2	3.00	0.079	green
α-terpinolene	+	−	−	22.82	1078.4	136.24	4.470	1.126	woody
Linalool	+	−	+	23.74	1092.1	154.3	2.970	0.016	citrus
Nonanal	+	+	+	23.98	1095.5	142.24	3.461	0.532	floral
Phenyethyl alcohol	+	−	−	24.35	1101.4	122.2	1.360	0.087	floral
α-campholenal	+	−	−	24.92	1113	152.24	2.587	0.415	green
2-ethylhexyl acetate	+	−	−	26.32	1146	172.27	3.686	0.413	earthy
Ethyl benzoate	+	−	−	27.45	1391	150.18	2.640	0.267	fruity
Decanal	+	+	−	29.38	1196.1	156.27	3.970	0.207	floral
Nerol	+	−	−	30.45	1218.6	154.25	3.47	0.013	floral
Geraniol	+	+	+	31.78	1246.9	154.25	3.56	0.021	floral
4-propylbenzaldehyde	+	−	−	32.34	1258.5	148.20	2.918	0.039	-
Decanol	+	−	+	32.65	1265	158.28	4.570	0.008	fatty
Vitispirane	+	−	−	32.83	1277	192.30	6.62	0.022	floral
Perillyl alcohol	+	−	+	33.72	1286	152.24	3.170	0.006	green
Eugenol	+	−	+	36.43	1350.7	164.20	2.27	0.010	spicy
Methyl acetate	−	−	+	35.03	1310	74.08	0.180	368.3	fruity
α-ionol	+	−	+	37.27	1371.2	194.32	4.492	0.001	floral
β-damascenone	+	−	−	37.48	1376.3	190.29	4.042	0.020	floral
Ethyl decanoate	+	+	−	38.09	1391	200.32	4.861	0.034	fruity
Dodecanal	+	−	−	38.34	1396.8	184.32	4.989	0.034	green
Trans-chariophyllene	−	+	−	38.50	1402	204.36	4.211	0.005	woody
α-ionone	+	−	+	38.83	1415.6	192.30	3.995	0.014	floral
Geranylacetone	+	+	+	39.50	1444.7	194.32	4.129	0.016	floral
β-ionone	+	+	+	40.29	1477.7	192.3	3.995	0.017	floral
Lilial	−	+	+	41.05	1513.6	204.31	4.216	0.005	floral
Myristaldehyde	+	+	−	42.48	1601	212.37	6.008	0.006	woody
Hexylcinnamal	+	+	−	44.34	1735.7	216.32	4.866	0.001	floral

RT—retention time of volatiles; RI—retention index of volatiles; MW—molecular weight; Log P—logarithm of octanol water coefficient that indicates the relative hydrophobicity of compound; VP—vapor pressure (mm/Hg); OD—odor descriptor (data were obtained from http://www.thegoodscentscompany.com and http://www.chemicalbook.com, accessed on 1 March 2023); + and −—volatile compounds were identified or not identified in tart cherry juice, protein matrix and formulated aggregates.

**Table 5:** Concentration of volatile compounds (µg/kg) in tart cherry juice (TC), dairy protein powder (Cas) and formulated aggregates (Cas2%_TC and Cas6%_TC).

Flavor Compounds	TC	Cas	Cas2%_TC	Cas6%_TC
**Aldehydes and Ketones**				
Hexanal	2.96 ± 0.06 ^a^	2.20 ± 0.03 ^b^	9.14 ± 0.71 ^c^	8.69 ± 0.86 ^c^
2-heptanone	0.19 ± 0.01	-	-	-
Heptanal	0.12 ± 0.02	-	-	-
Benzaldehyde	216.21 ± 2.53 ^d^	18.84 ± 0.15 ^a^	171.40 ± 2.72 ^b^	128.12 ± 4.79 ^c^
1-octen-3-one	4.69 ± 0.19	-	-	-
6-methyl-5-hepten-2-one	7.60 ± 0.27	-	-	-
Octanal	-	3.57 ± 0.07 ^a^	5.53 ± 0.33 ^b^	12.26 ± 1.66 ^c^
Nonanal	24.77 ± 0.23 ^c^	5.64 ± 0.08 ^a^	14.64 ± 0.29 ^b^	15.16 ± 0.16 ^b^
Decanal	9.84 ± 0.22 ^b^	3.55 ± 0.08 ^a^	-	-
4-propylbenzaldehyde	5.96 ± 0.28	-	-	-
Dodecanal	0.49 ± 0.05	-	-	-
Geranylacetone	6.38 ± 0.18 ^d^	1.54 ± 0.01 ^c^	1.28 ± 0.09 ^b^	0.96 ± 0.02 ^a^
Lillial	-	1.86 ± 0.01	1.77 ± 0.06	0.00 ± 0.00
Myristaldehyde	0.34 ± 0.01 ^a^	1.74 ± 0.01 ^b^	-	-
Hexylcinnamal	0.70 ± 0.01 ^a^	0.96 ± 0.01 ^b^	-	-
**Alcohols**				
Octen-3-ol	-	1.01 ± 0.01	-	-
2-ethyl-1-hexanol	3.99 ± 0.10 ^a^	14.46 ± 0.23 ^c^	4.34 ± 0.26 ^a^	6.06 ± 0.49 ^b^
Benzyl alcohol	48.55 ± 0.78 ^c^	-	34.48 ± 3.02 ^b^	23.60 ± 0.45 ^a^
1-octanol	-	5.88 ± 0.20 ^a^	11.01 ± 0.38 ^b^	12.62 ± 0.40 ^c^
Phenethyl alcohol	10.90 ± 0.35	-	-	-
Decanol	4.33 ± 0.00 ^a^	-	7.18 ± 0.76 ^b^	8.84 ± 0.95 ^b^
Perillyl alcohol	1.74 ± 0.08 ^b^	-	1.80 ± 0.20 ^b^	0.82 ± 0.22 ^a^
**Terpenes**				
D-limonene	108.42 ± 0.15 ^c^	-	63.56 ± 1.94 ^a^	92.21 ± 7.79 ^b^
α-terpinolene	2.33 ± 0.01	-	-	-
Linalool	14.22 ± 0.16 ^b^	-	13.56 ± 0.40 ^b^	8.70 ± 0.53 ^a^
α-campholenal	11.92 ± 0.04	-	-	-
Nerol	11.02 ± 0.11	-	-	-
Geraniol	0.81 ± 0.04 ^a^	2.09 ± 0.01 ^b^	2.25 ± 0.10 ^b^	0.65 ± 0.65 ^a^
Vitispirane	1.48 ± 0.13	-	-	-
Eugenol	6.25 ± 0.10 ^a^	-	11.63 ± 0.27 ^b^	5.16 ± 0.10 ^a^
α-ionol	39.84 ± 1.04 ^c^	-	32.74 ± 1.48 ^b^	15.18 ± 0.58 ^a^
Trans-chariophyllene	-	3.58 ± 0.03	-	-
β-damascenone	8.42 ± 0.26	-	-	-
α-ionone	1.63 ± 0.03 ^c^	-	0.59 ± 0.10 ^b^	0.00 ± 0.00 ^a^
β-ionone	6.13 ± 0.02 ^d^	0.66 ± 0.01 ^a^	2.20 ± 0.05 ^c^	1.49 ± 0.03 ^b^
**Esters**				
2-ethylhexyl acetate	0.40 ± 0.04	-	-	-
Ethyl benzoate	0.44 ± 0.04	-	-	-
Methyl acetate	-	-	83.05 ± 7.66 ^b^	78.38 ± 3.25 ^a^
Ethyl decanoate	3.40 ± 0.07 ^b^	0.05 ± 0.01 ^a^	-	-

Values in the same row labeled with different letters (^a–d^) were statistically different.

## Data Availability

Data is contained within the article.

## Supplementary Materials

The following supporting information can be downloaded at: https://www.mdpi.com/article/10.3390/foods12112104/s1, Figure S1: HPLC chromatogram at 520 nm of sample Cas2%_TC (1—cyanidin-3-glucosyl-rutinoside; 2—cyanidin-3-rutinoside); Figure S2 HPLC chromatogram at 360 nm of sample Cas2%_TC (3—rutin; 4—quercetin); Figure S3 HPLC chromatogram at 320 nm of sample Cas2%_TC (5—chlorogenic acid; 6—*p*-coumaric acid derivate; 7—neochlorogenic acid; 8—*p*-coumaric acid); Figure S4 HPLC chromatogram at 210 nm of sample Cas2%_TC (9—(−)-epicatechin).
