# Supplementary material for: Dairy-Protein-Based Aggregates as Additives Enriched with Tart Cherry Polyphenols and Flavor Compounds
Source: Foods. 2023 May 24;12(11):2104. doi: 10.3390/foods12112104 (PMC10252731; doi:10.3390/foods12112104)
Supplement: Supplementary file 1 [file foods-12-02104-s001.zip › foods-2416138-supplementary.pdf]

Supplementary material

Dairy Protein-Based Aggregates as Additives Enriched with Tart Cherry Polyphenols and Flavor Compounds

Mirela Kopjar [1,*], Ivana Buljeta [1], Ina Ćorković [1], Vanja Kelemen [2], Anita Pichler [1], Ivana Ivić [1] and Josip Šimunović[3]

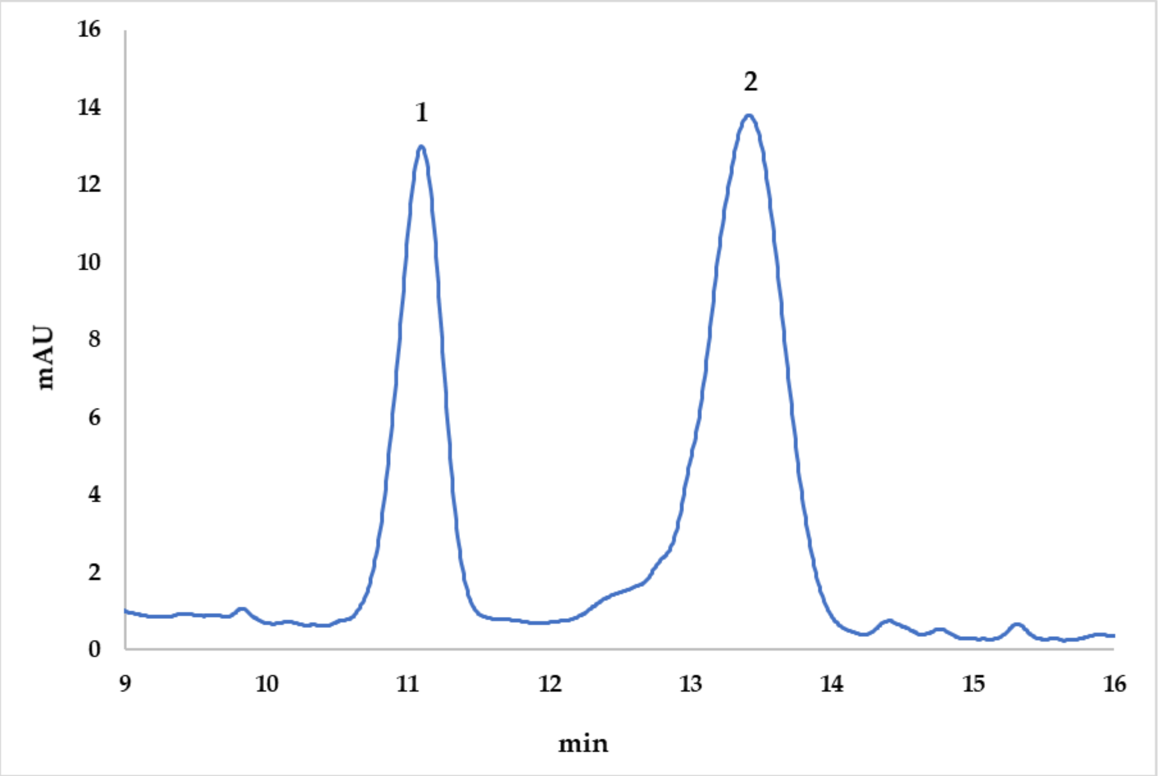

Figure S1 HPLC chromatogram at 520 nm of sample Cas2%_TC (1 – cyanidin-3-glucosyl-rutinoside; 2 – cyanidin-3-rutinoside)

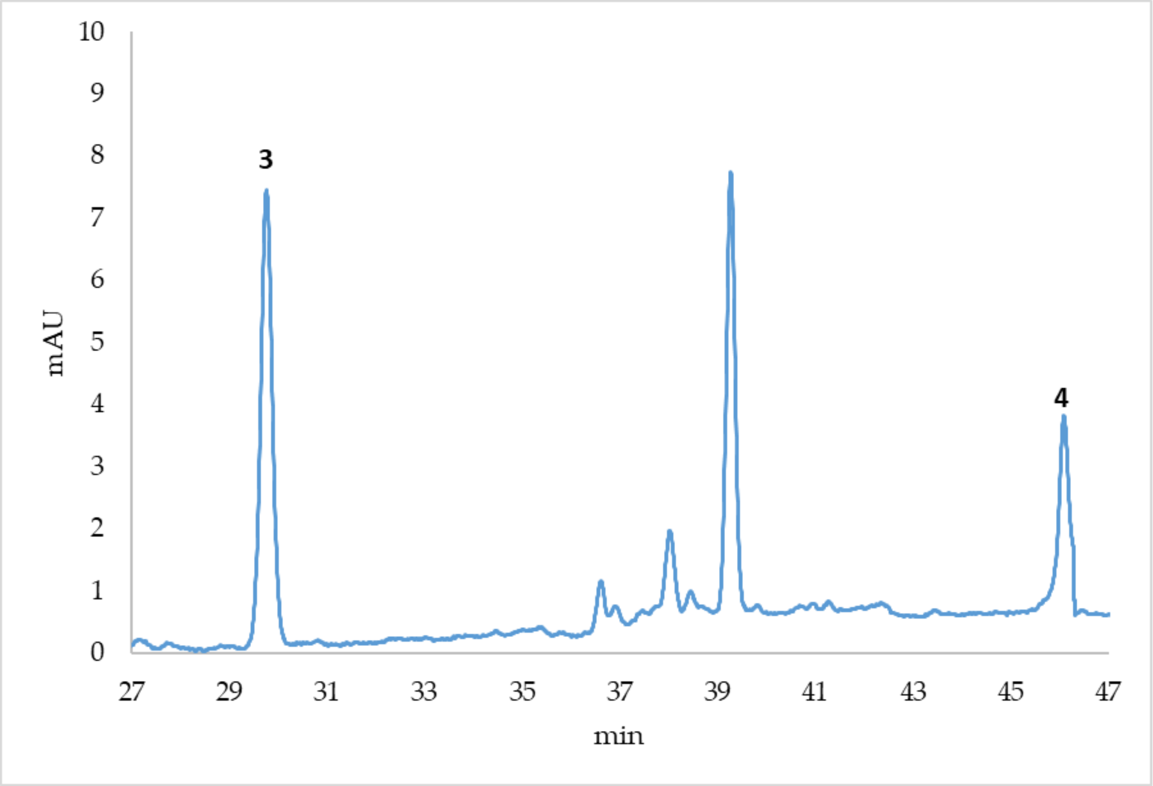

Figure S2 HPLC chromatogram at 360 nm of sample Cas2%_TC (3 – rutin; 4 – quercetin)

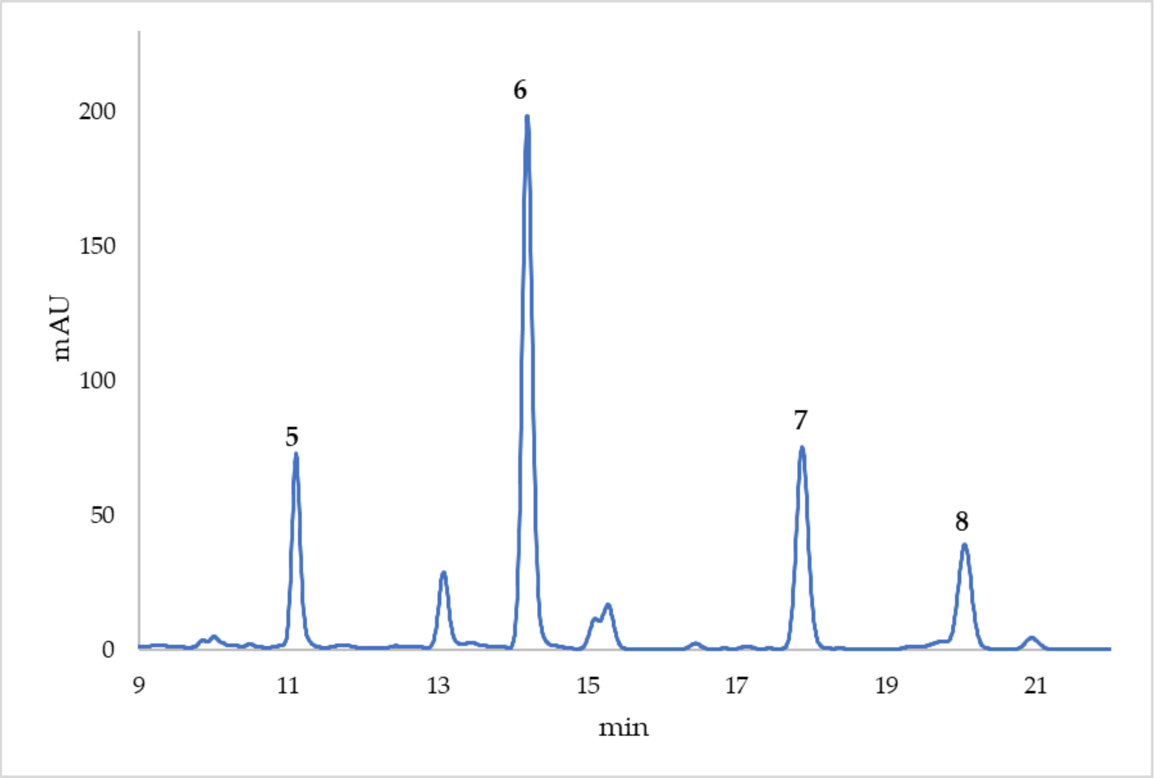

Figure S3 HPLC chromatogram at 320 nm of sample Cas2%_TC (5 – chlorogenic acid; 6 – *p*-coumaric acid derivate; 7 – neochlorogenic acid; 8 – *p*-coumaric acid)

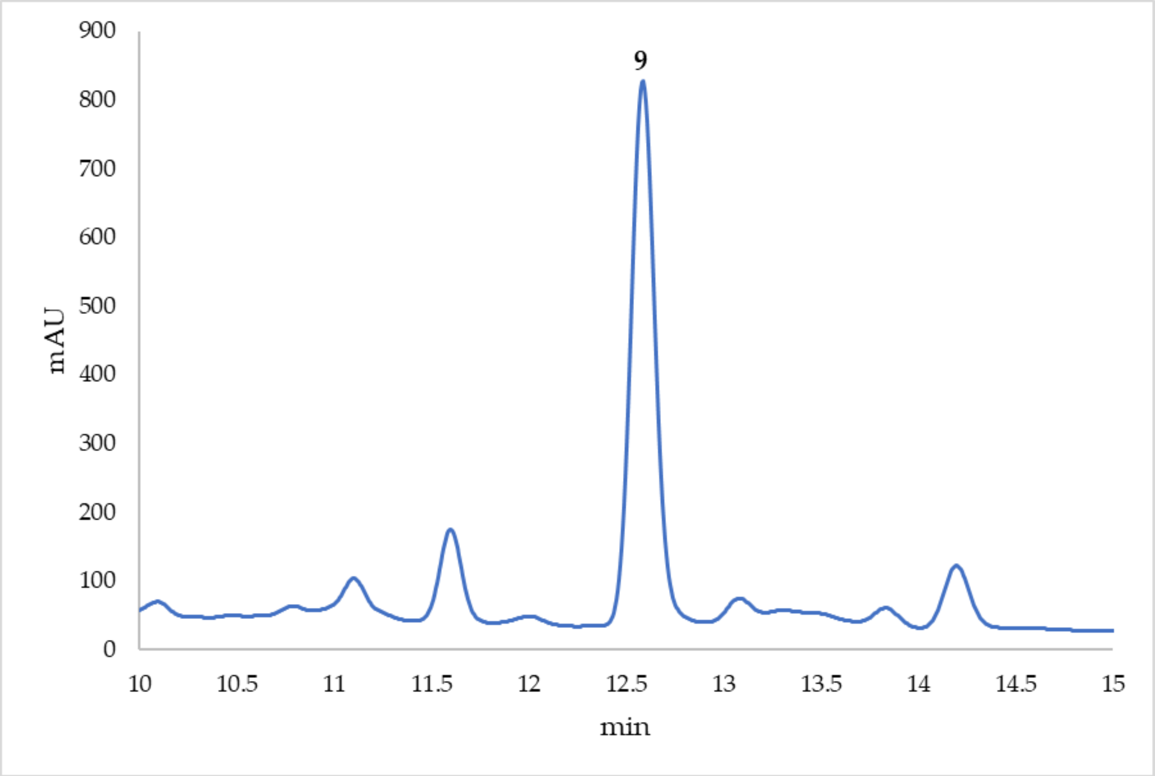

Figure S4 HPLC chromatogram at 210 nm of sample Cas2%_TC (9 – (-)-epicatechin)
